# Prospective Multicenter Evaluation of a Novel Low‐Axial‐Force Colonic Stent for Malignant Colorectal Obstruction

**DOI:** 10.1002/deo2.70466

**Published:** 2026-11-09

**Authors:** Ryo Otsuka, Shuji Saito, Naoki Fujimoto, Norihiro Hanabata, Akinari Takao, Kazuhiro Narita, Toshio Kuwai, Shungo Endo, Jun Omori, Toshiyuki Enomoto, Ryuichi Yamamoto, Yoshihiro Sasaki, Yoshihisa Saida, Hiroyuki Isayama

**Affiliations:** ^1^ Division of Surgery, Gastrointestinal Center Yokohama Shin‐Midori General Hospital Kanagawa Japan; ^2^ Department of Gastroenterology Tane General Hospital Osaka Japan; ^3^ Department of Gastroenterology and Oncology Aomori Prefectural Central Hospital Aomori Japan; ^4^ Department of Gastroenterology Tokyo Metropolitan Cancer and Infectious Diseases Center Komagome Hospital Tokyo Japan; ^5^ Department of Surgery Kawasaki Saiwai Hospital Kanagawa Japan; ^6^ Department of Gastroenterology National Hospital Organization, Kure Medical Center and Chugoku Cancer Center Hiroshima Japan; ^7^ Gastrointestinal Endoscopy and Medicine Hiroshima University Hospital Hiroshima Japan; ^8^ Department of Coloproctology, Aizu Medical Center Fukushima Medical University Fukushima Japan; ^9^ Department of Gastroenterology Nippon Medical School, Graduate School of Medicine Tokyo Japan; ^10^ Department of Surgery Toho University Ohashi Medical Center Tokyo Japan; ^11^ Department of Gastroenterology Tokyo‐West Tokushukai Hospital Tokyo Japan; ^12^ Department of Gastroenterology National Hospital Organization Disaster Medical Center Tokyo Japan; ^13^ Department of Gastroenterology, Graduate School of Medicine Juntendo University Tokyo Japan

**Keywords:** colorectal neoplasms, intestinal obstruction, palliative care, preoperative care, self‐expandable metallic stents

## Abstract

**Objectives:**

Self‐expandable metal stents (SEMSs) are used as a bridge to surgery (BTS) or for palliation (PAL) in malignant colorectal obstruction, but high axial force may contribute to perforation. We prospectively evaluated the efficacy and safety of the low‐axial‐force Naturfit colonic stent in both settings.

**Methods:**

This prospective multicenter single‐arm study enrolled 208 patients at 11 Japanese institutions between 2019 and 2022 (BTS, *n* = 137; PAL, *n* = 71). The primary endpoint was clinical success of colorectal stenting; secondary endpoints included technical success, adverse events, surgical outcomes after BTS stenting, and 12‐month palliative outcomes.

**Results:**

Technical success and clinical success of colorectal stenting were both 99.0%, with perforation in three patients (1.4%). In the BTS cohort, clinical success of BTS was 97.1%; no perforations occurred, and early and late adverse‐event rates were 1.5% and 3.7%. All patients underwent elective surgery; colostomy and laparoscopic surgery rates were 4.4% and 87.6%. In the palliative cohort, clinical success of colorectal stenting was 98.6%, late adverse events occurred in 16.9%, and 12‐month overall survival and stent patency were 46.6% and 67.6%.

**Conclusions:**

Naturfit achieved high technical and clinical success of colorectal stenting with a low perforation rate. These findings support its feasibility for malignant colorectal obstruction in both BTS and palliative settings, although comparative studies are needed to determine relative advantages over other SEMSs.

**Trial Registration:**

University Hospital Medical Information Network Clinical Trial Registry: UMIN000035224

## 1 Introduction

Malignant colorectal obstruction occurs in 8%–14% of patients with colorectal cancer [1, 2, 3] and requires urgent decompression. Emergency surgery, often including colostomy, is associated with less favorable outcomes than elective surgery [4, 5]. Colonic self‐expandable metal stents (SEMSs) are therefore used for palliation (PAL) or as a bridge to surgery (BTS), as recommended by the European Society of Gastrointestinal Endoscopy (ESGE) [6]. Stent‐related perforation is associated with adverse oncologic outcomes, including increased locoregional recurrence and poorer relapse‐free survival [7, 8]. Besides bevacizumab use and placement for benign lesions, stent design is a recognized risk factor [9]. Mechanical properties include radial force, which expands the stent, and axial force (AF), which straightens a bent stent [10, 11].

HANAROSTENT Naturfit (M.I. Tech Co., Ltd., Republic of Korea; distributed by Boston Scientific Japan) was designed with lower AF than WallFlex to improve conformability in curved bowel and incorporates radiopaque markers and a repositionable delivery system. Naturfit is currently available only in Japan. We therefore conducted a prospective multicenter single‐arm study to descriptively evaluate its efficacy and safety in patients with malignant colorectal obstruction. The study was not designed to demonstrate superiority over other colonic SEMSs.

## 2 Materials and Methods

### 2.1 Study Design

This prospective multicenter single‐arm observational study was conducted at 11 Japanese institutions to descriptively evaluate the efficacy and safety of Naturfit. Institutional review board approval was obtained at each site, and the study was registered in the University Hospital Medical Information Network Clinical Trial Registry (UMIN000035224). Eligible patients had malignant colorectal obstruction requiring colonic stenting. Exclusion criteria were bowel ischemia, suspected or impending perforation, intra‐abdominal abscess, severe organ dysfunction, inability to undergo endoscopy, or previous colonic stenting. The choice of SEMS was not specified by the protocol and was determined by each institution and endoscopist in routine practice. Patients scheduled for Naturfit placement were prospectively assessed for eligibility and provided written informed consent before the procedure. Clinical data were collected prospectively in an electronic registry, although entry could occur shortly after stent placement. Patients scheduled for resection comprised the BTS cohort, and those without planned resection comprised the PAL cohort; follow‐up continued until postoperative discharge for BTS and 12 months after stent placement for PAL.

### 2.2 Stent Placement

Stent placement followed the JCSSPRG technical guidelines. A colonoscope was advanced to the obstruction, and a guidewire and catheter were passed across the stenosis under fluoroscopy; contrast was injected to assess stricture length. Clip marking was recommended, balloon dilation was prohibited, and the stent was deployed using the through‐the‐scope technique under fluoroscopic guidance.

Only uncovered Naturfit stents were used. Naturfit is designed with low AF to enhance conformability in curved colonic segments (Figure 1A,B), with radiopaque markers and a repositionable delivery system. Delivery systems were 9 or 10 Fr; stent diameters were 18, 20, or 22 mm and lengths were 6, 9, or 12 cm.

**FIGURE 1:**
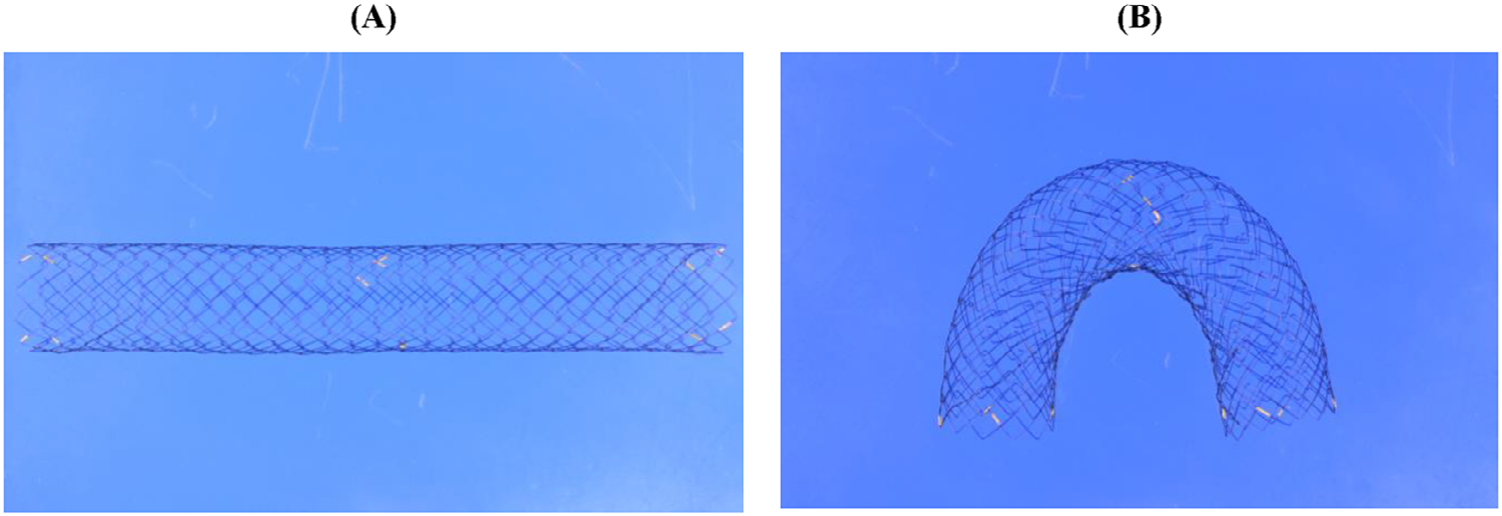
Photographs of the Naturfit colonic stent. (A) The deployed stent in a straight configuration. (B) The stent in a curved configuration, illustrating its conformability. Radiopaque markers are visible on the stent.

### 2.3 Data Collection

Clinical data were collected prospectively in an electronic registry. Baseline variables included demographics, primary disease and stage, Eastern Cooperative Oncology Group (ECOG) performance status, American Society of Anesthesiologists Physical Status classification, CROSS score, symptoms, and laboratory findings. Procedural variables included stenosis characteristics, stent number and size, procedure time, and technical success. Adverse events were classified as early (≤7 days) or late (≥8 days). Surgical, pathological, and postoperative outcomes were collected for BTS, and survival and post‐stenting chemotherapy for PAL. CROSS grades oral intake from 0 (continuous decompression required) to 4 (full diet without obstructive symptoms) [12].

### 2.4 Clinical Outcomes

The primary endpoint was clinical success of colorectal stenting, defined as improvement of obstructive symptoms with radiologic decompression within 24 h. In the BTS cohort, clinical success of BTS was defined as sustained decompression until elective surgery without stent‐related adverse events, endoscopic reintervention, or emergency surgery. Adverse events judged to be unrelated to the stent were recorded as adverse events after stent placement but did not constitute failure of clinical success of BTS. Secondary endpoints were technical success, stent‐related adverse events (early, ≤7 days; late, ≥8 days), perioperative outcomes and one‐stage anastomosis in BTS, and stent patency in PAL. Technical success was defined as deployment across the target stenosis in one attempt.

### 2.5 Statistical Analysis

Because prior data were insufficient for a formal calculation, the target sample size was set at 200 based on feasibility during the study period. Analyses were descriptive and stratified by indication (BTS or PAL). Continuous variables are presented as mean ± standard deviation or median (interquartile range), and categorical variables as counts and percentages; binary outcomes include 95% confidence intervals (CIs). In PAL, 12‐month overall survival and stent patency were estimated using the Kaplan–Meier method and reported at 365 days with 95% CIs. For the stent patency analysis, patients who died while the stent remained patent were censored at the date of death. Patients without loss of stent patency were otherwise censored at the last follow‐up or at 365 days, whichever came first. No formal comparisons between BTS and PAL were planned. Missing data were analyzed by complete case with denominators reported for each outcome. Patients lost to follow‐up were censored at the date last known alive. Analyses used R (R Foundation for Statistical Computing, Vienna, Austria), and reporting followed the STROBE guidelines for cohort studies.

ChatGPT (OpenAI) was used to assist with English‐language editing, manuscript organization, and wording during manuscript revision. All artificial intelligence‐assisted output was reviewed and edited by the authors, who take full responsibility for the final content.

## 3 Results

### 3.1 Patient Characteristics

Patient flow is summarized in Figure 2. A total of 208 patients were enrolled between March 2019 and January 2022, and all were included in the analysis. Baseline characteristics are shown in Table 1. The mean age was 74.3 years, 73.5% of patients had an ECOG PS of 0–1, and primary colorectal cancer accounted for 97.1% of obstructions. Obstruction involved the right‐sided colon in 31.7%, left‐sided colon in 50.5%, and rectum in 17.8%; 91.8% had complete obstruction. Treatment intent was BTS in 137 patients (65.9%) and PAL in 71 (34.1%).

**FIGURE 2:**
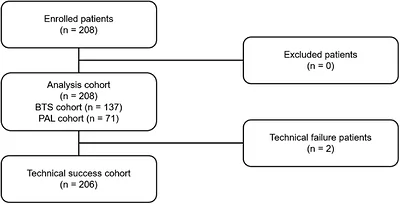
Patient flow of the study cohort. Of the 208 patients enrolled, 137 were treated as bridge to surgery (BTS) and 71 for palliation (PAL).

**TABLE 1:** Baseline patient characteristics (*n* = 208).

**Age, years, mean ± SD**	74.3 ± 11.3
**Sex, *n* (%)**	
Male	123 (59.1)
Female	85 (40.9)
**Height, cm, mean ± SD**	159.2 ± 9.8
**Body weight, kg, mean ± SD**	53.1 ± 11.4
**Body mass index, kg/m^2^, mean ± SD**	20.8 ± 3.6
**ECOG PS, *n* (%)**	
0	62 (29.8)
1	91 (43.8)
2	35 (16.8)
3	19 (9.1)
4	1 (0.5)
**ASA‐PS classification, *n* (%)**	
1	42 (20.2)
2	131 (63.0)
3	32 (15.4)
4	3 (1.4)
**Any symptoms of obstruction, *n* (%)**	
Abnormal bowel movements	155 (74.5)
Abdominal pain	139 (66.9)
Bloating	135 (64.9)
Nausea	88 (42.3)
Vomiting	63 (30.3)
**Etiology of colorectal obstruction, *n* (%)**	
Primary colorectal cancer	202 (97.1)
Others	6 (2.9)
**Therapeutic intent, *n* (%)**	
Bridge to surgery	137 (65.9)
Palliation	71 (34.1)
**Obstruction/tumor site, *n* (%)**	
Right‐sided colon	66 (31.7)
Left‐sided colon	105 (50.5)
Rectum	37 (17.8)
**Complete/incomplete obstruction, *n* (%)**	
Complete obstruction	191 (91.8)
Incomplete obstruction	17 (8.2)
**Bowel decompression before stent placement, *n* (%)**	
None	190 (91.3)
Nasogastric tube	7 (3.4)
Transnasal ileus tube	2 (1.0)
Transanal ileus tube	9 (4.3)

### 3.2 Overall Clinical Outcomes of Stent Placement

#### 3.2.1 Technical Success

Technical success was achieved in 206 patients (99.0%; Table 2). The two technical failures were cecal perforation due to insufflation and misidentification of the target stenosis because of an additional tumor. A second stent was subsequently placed across the actual stenosis in the latter patient. Procedural and stent characteristics are summarized in Table 2.

**TABLE 2:** Overall clinical outcomes of stent placement (*n* = 208).

**Technical success, *n* (%) [95% CI]**	206 (99.0) [96.6–99.9]
**Technical failure, *n* (%)**	2 (1.0)
Perforation of the cecum due to insufflation	1 (0.5)
Misdiagnosis of stenosis due to another tumor	1 (0.5)
**Procedure time, min, mean ± SD**	30.9 ± 15.8
**Stricture length, cm, mean ± SD**	4.4 ± 2.1
**Strictures and stents placed, *n* (%)**	
Single stricture with one stent	204 (98.1)
Double stricture with two stents	3 (1.4)
Quadruple stricture with two stents	1 (0.5)
**Stent diameter, *n* (%)**	
First stent	
18 mm	1 (0.5)
20 mm	4 (1.9)
22 mm	203 (97.6)
Second stent	
22 mm	4 (100.0)
**Stent length, *n* (%)**	
First stent	
6 cm	58 (27.9)
9 cm	104 (50.0)
12 cm	46 (22.1)
Second stent	
6 cm	1 (25.0)
9 cm	2 (50.0)
12 cm	1 (25.0)
**Clinical success of colorectal stenting, *n* (%) [95% CI]**	206 (99.0) [96.6–99.9]
**Clinical failure, *n* (%)**	2 (1.0)
Perforation of the cecum due to insufflation	1 (0.5)
Misdiagnosis of stenosis due to another tumor	1 (0.5)

Table 2 summarizes characteristics and outcomes up to and including the stent placement procedure.

### 3.3 Clinical Success of Colorectal Stenting

Clinical success of colorectal stenting was achieved in 206 patients (99.0%; Table 2). Both clinical failures occurred in the two patients with technical failure described above.

### 3.4 CROSS Score before and After Stent Placement

The CROSS distribution shifted after stent placement, with score 0 decreasing from 95 patients (45.7%) to two (1.0%) and score 4 increasing from three (1.4%) to 201 (96.6%) (Table 3).

**TABLE 3:** CROSS score before and after stent placement (*n* = 208).

CROSS	Before, *n* (%)	After, *n* (%)
0	95 (45.7)	2 (1.0)
1	49 (23.6)	0 (0.0)
2	39 (18.8)	2 (1.0)
3	22 (10.6)	3 (1.4)
4	3 (1.4)	201 (96.6)

### 3.5 Clinical Outcomes in the BTS Cohort

#### 3.5.1 Clinical Success of BTS

In the BTS cohort, clinical success of BTS was achieved in 133 of 137 patients (97.1%; Table 4). Failures comprised one technical failure, two stent migrations, and one stent occlusion.

**TABLE 4:** Clinical outcomes in the bridge to surgery (BTS) cohort (*n* = 137).

**Clinical success of BTS, *n* (%) [95% CI]**	133 (97.1) [92.7–99.2]
**Early adverse events after stent placement, *n* (%) [95% CI]**	2 (1.5) [0.2–5.2]
Bleeding, *n*	1
DIC, *n*	1
**Late adverse events after stent placement, *n* (%) [95% CI]**	5 (3.7) [1.2–8.3]
Stent migration, *n*	2
Stent occlusion, *n*	1
Fever, *n*	2
**Days from stent placement to surgery, median (IQR), days**	22 (17–31)
**Scheduled elective surgery, *n* (%)**	137 (100.0)
**Surgical approach, *n* (%)**	
Laparoscopy	120 (87.6)
Open conversion from laparoscopy	7 (5.1)
Open surgery	10 (7.3)
**One‐stage anastomosis, *n* (%)**	135 (98.5)
**Colectomy, *n* (%)**	136 (99.2)
**Colostomy, *n* (%)**	6 (4.4)
**Postoperative complications, *n* (%)**	13 (9.5)
**Anastomotic leakage**	3 (2.2)
**Requiring reoperation**	2 (1.5)
**Bowel obstruction**	6 (4.4)
Requiring reoperation	2 (1.5)
**Intra‐abdominal abscess requiring drainage/reoperation**	1 (0.7)
**Other postoperative complications** ^a^	5 (3.6)
**Postoperative death, *n* (%)**	0 (0.0)

Values are presented as *n* (%) unless otherwise indicated. Early adverse events were defined as events occurring within 7 days after stent placement, and late adverse events were defined as events occurring thereafter until surgery. Confidence intervals are shown for the main binary outcomes.

a* Other postoperative complications included urinary tract infection (*n* = 1), lower gastrointestinal bleeding (*n* = 2), cerebral infarction (*n* = 1), and urinary retention (*n* = 1). Some patients experienced more than one postoperative complication.

### 3.6 Adverse Events After Stent Placement

Early adverse events occurred in two patients (1.5%; bleeding and DIC), both managed conservatively. Late adverse events occurred in five (3.7%): migration in two, occlusion in one, and fever in two. Bleeding, DIC, and fever were judged unrelated to the stent and therefore did not constitute BTS clinical failure. One migration occurred toward the oral side after day 8 in a 6‐cm splenic‐flexure stricture treated with a 22‐mm × 12‐cm stent; the other occurred toward the anal side on day 11 in a 7‐cm ascending‐colon stricture treated with the same stent size. Neither required additional decompression or emergency intervention. The stent occlusion occurred during bowel preparation and resolved conservatively.

### 3.7 Perioperative Outcomes

The median time to surgery was 22 days, and all patients underwent elective surgery. Laparoscopy was completed in 120 patients (87.6%), one‐stage anastomosis in 135 (98.5%), and colostomy was created in six (4.4%). Postoperative complications occurred in 13 patients (9.5%), including anastomotic leakage in three (two requiring reoperation), bowel obstruction in six (two requiring reoperation), and intra‐abdominal abscess in one; other complications are shown in Table 4. There were no stent‐related or postoperative deaths.

### 3.8 Clinical Outcomes in the PAL Cohort

#### 3.8.1 Clinical Success of Colorectal Stenting, Early Adverse Events, and Post‐Stenting Treatment

In the PAL cohort, clinical success of colorectal stenting was achieved in 70 of 71 patients (98.6%; Table 5). Early adverse events occurred in six patients (8.5%): perforation in one, stent occlusion in one, fever in three, and ileus in one. The perforation occurred in the patient with technical failure described above, who underwent emergency surgery and died on day 16. The stent occlusion was caused by fecal impaction and was treated by endoscopic cleaning.

**TABLE 5:** Clinical outcomes in the palliation (PAL) cohort within 12 months after stent placement (*n* = 71).

**Clinical success of colorectal stenting, *n* (%) [95% CI]**	70 (98.6) [92.4–100.0]
**Early adverse events after stent placement, *n* (%) [95% CI]**	6 (8.5) [3.2–17.5]
Stent occlusion, *n*	1
Perforation, *n*	1
Fever, *n*	3
Ileus, *n*	1
**Late adverse events within 12 months, *n* (%) [95% CI]**	12 (16.9) [9.0–27.7]
Stent occlusion, *n*	8
Stent migration, *n*	2
Perforation, *n*	2
Fever, *n*	3
Bleeding, *n*	1
**Treatment for primary malignancy after stent placement, *n* (%)**	
None	47 (66.2)
Chemotherapy	24 (33.8)
Use of molecular‐targeted agents	9 (12.7)
Bevacizumab‐containing treatment	4 (5.6)
Radiotherapy	0 (0.0)
**Kaplan–Meier endpoints within 12 months after stent placement**	
Overall survival, median, days	300
Deaths within 12 months, *n*	33
12‐month overall survival, % [95% CI]	46.6 [33.8–58.5]
Stent patency, median, days	Not reached
Stent patency events within 12 months, *n*	12
12‐month stent patency, % [95% CI]	67.6 [46.2–82.1]

Values are presented as *n* (%) unless otherwise indicated. Early adverse events were defined as events occurring within 7 days after stent placement, and late adverse events were assessed thereafter up to 12 months. Some patients experienced more than one late adverse event. Overall survival and stent patency were estimated using the Kaplan–Meier method.

Molecular‐targeted agents and bevacizumab‐containing treatment were included within the chemotherapy group.

After stent placement, 24 patients (33.8%) received chemotherapy, including nine treated with molecular‐targeted agents and four with bevacizumab‐containing regimens. No perforation occurred among the four bevacizumab‐treated patients; one developed bleeding requiring surgery, and one developed stent occlusion. No patient received radiotherapy.

### 3.9 Late Adverse Events and Time‐to‐Event Outcomes

Late adverse events within 12 months occurred in 12 of 71 patients (16.9%), with 16 events in total; stent occlusion was the most common event (*n* = 8) (Table 5). Most stent occlusions were managed by stent‐in‐stent placement; two patients ultimately required surgery for reocclusion or bleeding. Two late perforations occurred: one on day 214 in a 4‐cm sigmoid‐colon stricture treated with a 22‐mm × 9‐cm stent, requiring Hartmann's procedure, and the other was a small‐intestinal perforation on day 40 in a 5‐cm ileocecal stricture treated with the same stent size and was managed conservatively. Stent flexion was not prospectively recorded in either case. Overall, four patients (5.6%) required surgery after stent placement.

Median overall survival was 300 days, whereas median stent patency was not reached. The 12‐month overall survival and stent patency rates were 46.6% (95% CI, 33.8–58.5) and 67.6% (95% CI, 46.2–82.1), respectively (Table 5; Figures 3 and 4).

**FIGURE 3:**
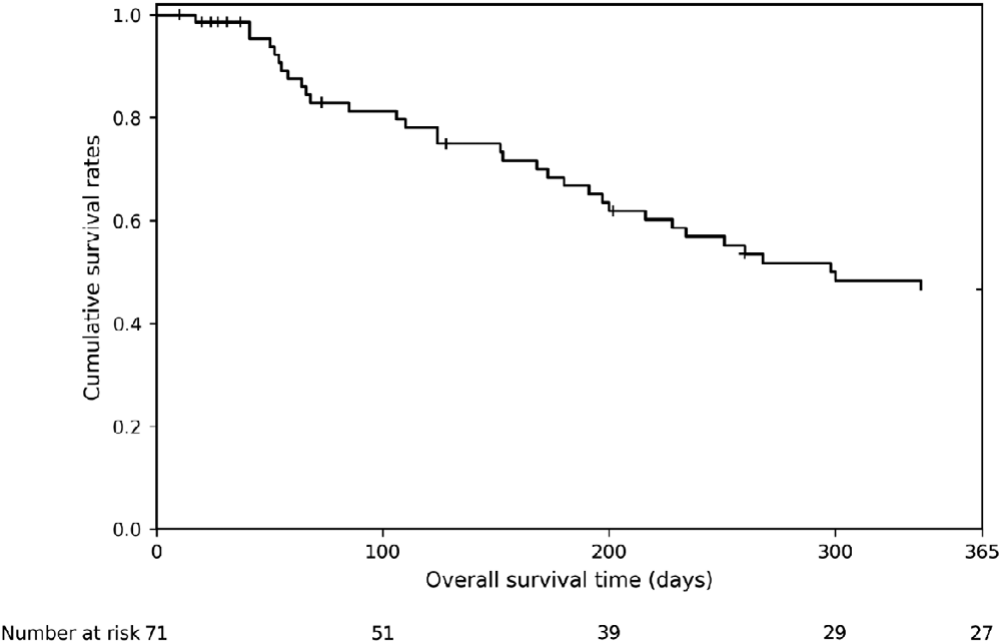
Kaplan–Meier curve for overall survival in the palliation (PAL) cohort within 12 months after stent placement. Overall survival was evaluated in the PAL cohort (*n* = 71) within 12 months after stent placement. Death was defined as the event, and censored observations are indicated by “+”. Numbers at risk are shown at 100‐day intervals. Confidence bands are not shown.

**FIGURE 4:**
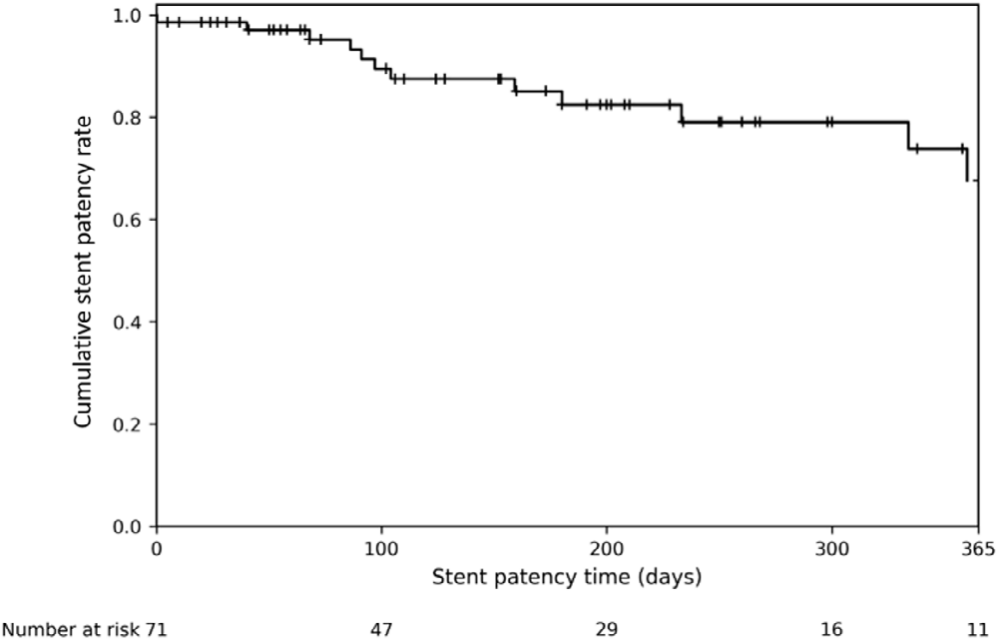
Kaplan–Meier curve for stent patency in the palliation (PAL) cohort within 12 months after stent placement. Stent patency was evaluated in the PAL cohort (*n* = 71) within 12 months after stent placement. Loss of stent patency was defined as the event, and censored observations are indicated by “+”. Numbers at risk are shown at 100‐day intervals. Confidence bands are not shown.

## 4 Discussion

This prospective multicenter single‐arm study evaluated the efficacy and safety of Naturfit. Perforation occurred in three of 208 patients (1.4%, 95% CI 0.3–4.2), numerically lower than the 3%–7.5% reported for conventional colonic stents [9, 13, 14]. A retrospective study of 73 patients reported a perforation rate of 4.1% and found no significant predictors of stent‐related complications [15]. However, differences in patient selection, procedural techniques, and study periods limit direct comparison, and this study was not designed to demonstrate superiority over other stents.

Naturfit was designed with lower AF than WallFlex to improve conformability in curved bowel. Sasaki et al. defined the AF zero‐border angle as the angle at which stent torque almost disappeared during stretching. This angle was 5° for the 22‐mm WallFlex stent and 63° for the 22‐mm Naturfit stent, indicating that Naturfit's AF diminished while the stent remained substantially bent, whereas that of WallFlex persisted until the stent was nearly straight. This property may reduce sustained stress on the intestinal wall in curved segments [10]. However, this single‐arm study cannot establish that the low perforation rate was attributable specifically to the low‐axial‐force design.

Procedural factors may also have contributed to the favorable outcomes. Stent placement followed standardized techniques under JCSSPRG guidance, including careful evaluation of the stenotic segment, guidewire passage, fluoroscopic deployment, and avoidance of balloon dilation. Maturation and standardization of colonic stenting over time may also have influenced the results. Thus, the low perforation rate may reflect a combination of device characteristics, operator expertise, standardized procedural practice, and temporal advances. Previous prospective studies from our group reported technical success rates of 97.8%–99.5% and clinical success rates of colorectal stenting of 92.1%–97.0% [16, 17, 18]. In the present study, both technical success and clinical success of colorectal stenting were 99.0%, and clinical success of BTS was 97.1%. Selected prospective multicenter studies of other colonic SEMSs are summarized in Table 6 for descriptive comparison [19].

**TABLE 6:** Treatment outcomes in selected prospective multicenter studies of colonic self‐expandable metal stents (SEMSs).

Study	Stent	Treatment intent	*n*	Technical success, %	Clinical success, %	Perforation, %
Saito et al. [16]	WallFlex	BTS	312	97.8	92.1	1.6
Ishibashi et al. [12]	WallFlex	PAL	200	98.5	94.5	7.0
Murakami et al. [18]	JENTLLY	BTS/PAL	199	99.5	97.0	0.5
Mizumoto et al. [19]	Niti‐S MD/BB	BTS/PAL	193	98.4	95.9	2.1
Present study	Naturfit	BTS/PAL	208	99.0	99.0	1.4

Data are presented for descriptive contextual comparison only. Differences in patient selection, treatment intent, endpoint definitions, and follow‐up duration preclude direct comparison between studies. Definitions of clinical success differed among the included studies. BTS, bridge to surgery; PAL, palliation; SEMSs, self‐expandable metal stents.

Naturfit's repositionability during deployment and highly visible radiopaque markers may facilitate accurate stent positioning. However, their specific contribution to the low perforation rate cannot be determined from this single‐arm study. Late stent migration occurred in four patients overall (1.9%; 95% CI, 0.5–4.9), including two in the BTS cohort and two in the PAL cohort. A 2016 systematic review reported a migration rate of 6.4% [20], and stent design, size mismatch, and placement in curved segments have been reported as risk factors [9]. The two BTS migrations occurred in opposite directions and at different anatomical locations, with no common mechanism identified. Insufficient anchoring related to lesion configuration, the relative position of the stenosis within the stent, or mild luminal narrowing may have contributed; however, these factors were not prospectively recorded in sufficient detail and remain speculative. The contribution of Naturfit's mechanical properties to these migration events could not be determined.

In the BTS cohort, all patients proceeded to elective surgery. Emergency surgery is associated with less favorable short‐term surgical outcomes than stent‐based BTS [21]. The colostomy rate in the present study was 4.4%, numerically lower than the temporary and permanent stoma rates of 33.9% and 22.2%, respectively, reported after stent‐based BTS in a previous meta‐analysis [5]. Postoperative complications occurred in 9.5%, including anastomotic leakage in 2.2% and bowel obstruction in 4.4%, with no postoperative deaths. Previous studies have reported lower postoperative morbidity with stent‐based BTS than with emergency surgery [5, 21]. However, without a concurrent emergency‐surgery control group, these outcomes should be interpreted descriptively and cannot be attributed to Naturfit.

In the PAL cohort, clinical success of colorectal stenting was achieved in 98.6%, and late adverse events occurred in 16.9%. Because median overall survival was 300 days and the 12‐month overall survival rate was 46.6%, a substantial proportion of patients died before completing 12 months of follow‐up. Therefore, death acted as a competing event for late stent‐related adverse events, and the observed 12‐month adverse‐event rate may underestimate the cumulative risk among patients surviving throughout follow‐up.

Four patients (5.6%) required surgery. Previous studies reported clinical success rates of 85.0%–89.6%, complication rates of 18.3%–38.0%, and subsequent surgery rates of 27.1%–29.4% [22, 23]. However, direct comparison is limited by differences in patient characteristics, including performance status and disease burden, and by historical changes in stenting techniques and patient management. Therefore, the present findings should not be interpreted as evidence of superior safety or efficacy of Naturfit. Stent occlusion was the most common complication (11.3%). Stent occlusion has been reported as an important long‐term complication after palliative colonic stenting [23, 24]. Most stent occlusions were managed with stent‐in‐stent placement; however, two patients ultimately required surgery for reocclusion or bleeding.

Late perforation occurred in two patients (2.8%). One underwent Hartmann's procedure, whereas the other was managed conservatively. Stent flexion was not prospectively recorded in either case, and its contribution could not be determined. One patient had received chemotherapy after stent placement but no molecularly targeted agent, whereas the other had received no chemotherapy; neither received bevacizumab. Bevacizumab‐containing chemotherapy has been associated with an increased risk of perforation after colonic stenting [6, 9, 25]. Among 24 patients (33.8%) receiving chemotherapy, four received bevacizumab‐containing regimens. No perforation occurred among these four patients; one developed bleeding requiring surgical resection for hemostasis, and one developed stent occlusion. The interval between stent placement and bevacizumab initiation was unavailable in the study dataset. These findings should be interpreted cautiously because of the small number of patients receiving bevacizumab. The high clinical success rate and observed 12‐month stent patency suggest that Naturfit provided effective decompression in the PAL cohort. However, these findings should be interpreted in light of patient selection, competing mortality, and the absence of a concurrent comparator. Careful follow‐up remains warranted because late stent occlusion and perforation may occur.

### 4.1 Strengths and Limitations

The strengths of this study include its prospective multicenter design, standardized techniques across participating centers, and detailed evaluation of both BTS and PAL outcomes. Several limitations should be acknowledged. First, the single‐arm design without a comparator precludes assessment of the relative efficacy or safety of Naturfit. Second, SEMS selection was determined by each institution and endoscopist, and Naturfit was not used in all consecutive patients; therefore, device‐selection bias may limit generalizability. Third, BTS and PAL assignment was based on treatment intent rather than randomization, and differences in patient and disease characteristics may have confounded comparisons between cohorts. Fourth, patient selection and historical changes in stenting techniques and supportive care may limit comparisons of the PAL cohort with previous studies. Fifth, quality of life was not assessed, precluding evaluation of the patient‐reported impact of the low colostomy rate. Finally, death competed with late adverse events in the PAL cohort, and because no formal competing‐risk analysis was performed, the 12‐month adverse‐event rate represents observed incidence rather than cumulative risk independent of death.

### 4.2 Clinical Implications and Conclusions

Naturfit incorporates design features intended to enhance conformability during colonic stenting. In this prospective multicenter single‐arm study, technical and clinical success of colorectal stenting were high, with a low perforation rate. All BTS patients underwent elective surgery with a low colostomy rate, while PAL patients achieved effective decompression with acceptable 12‐month outcomes. These findings should be interpreted in light of standardized procedural techniques, the absence of a concurrent comparator, and the risk of late adverse events.

## Author Contributions

**Conception and design**: Shuji Saito, Hiroyuki Isayama, and Yoshihisa Saida. **Analysis and interpretation of the data**: Ryo Otsuka and Shuji Saito. **Drafting of the article**: Ryo Otsuka. **Critical revision of the article for important intellectual content**: Shuji Saito, Yoshihiro Sasaki, Hiroyuki Isayama, and Yoshihisa Saida. **Data curation**: Ryo Otsuka, Shuji Saito, Naoki Fujimoto, Norihiro Hanabata, Akinari Takao, Kazuhiro Narita, Toshio Kuwai, Shungo Endo, Jun Omori, Toshiyuki Enomoto, Ryuichi Yamamoto, Yoshihiro Sasaki, Yoshihisa Saida, and Hiroyuki Isayama. **Final approval of the article**: All authors.

## Funding

This study was conducted by the Japan Colorectal Stent Safety Procedure Research Group, which is affiliated with the Japan Gastroenterological Endoscopy Society. The study was supported by the Japan Colorectal Stent Safety Procedure Research Group through subsidies from the Japan Gastroenterological Endoscopy Society, annual membership fees from individual and supporting members, and research donations from contributing companies. The funders had no role in study design, data collection, analysis, interpretation, manuscript preparation, or the decision to submit the manuscript for publication.

## Ethics Statement

**Approval of the research protocol by an Institutional Review Board**: The institutional review boards of all participating hospitals approved the study before its initiation. The study was conducted in accordance with the principles of the Declaration of Helsinki.

## Consent

Written informed consent was obtained from all patients before stent placement.

## Conflicts of Interest

Hiroyuki Isayama has received research grants and speaker honoraria from Boston Scientific Japan Co., Ltd., and speaker honoraria from SB‐KAWASUMI LABORATORIES, INC. The other authors declare no financial relationships or conflicts of interest. Individual conflicts of interest for the researchers were managed and disclosed prior to the study in accordance with the regulations of each participating medical institution.

## Data Availability

The data that support the findings of this study are available from the corresponding author upon reasonable request. The data are not publicly available because of privacy and ethical restrictions.
